# S100A8 and S100A9 Promote Apoptosis of Chronic Eosinophilic Leukemia Cells

**DOI:** 10.3389/fimmu.2020.01258

**Published:** 2020-08-06

**Authors:** Ji-Sook Lee, Na Rae Lee, Ayesha Kashif, Seung-Ju Yang, A. Reum Nam, Ik-Chan Song, Soo-Jung Gong, Min Hwa Hong, Geunyeong Kim, Pu Reum Seok, Myung-Shin Lee, Kee-Hyung Sung, In Sik Kim

**Affiliations:** ^1^Department of Clinical Laboratory Science, Wonkwang Health Science University, Iksan, South Korea; ^2^Department of Biomedical Laboratory Science, Eulji University School of Medicine, Daejeon, South Korea; ^3^Department of Senior Healthcare, BK21 Plus Program, Graduate School, Eulji University, Daejeon, South Korea; ^4^Department of Biomedical Laboratory Science, Konyang University, Daejeon, South Korea; ^5^Department of Biochemistry, BK21 Plus and Research Institute for Veterinary Science, School of Veterinary Medicine, Seoul National University, Seoul, South Korea; ^6^Division of Hematology/Oncology, Department of Internal Medicine, Chungnam National University School of Medicine, Chungnam National University Hospital, Daejeon, South Korea; ^7^Department of Internal Medicine, Eulji Medical Center, Eulji University School of Medicine, Daejeon, South Korea; ^8^Department of Microbiology and Immunology, Eulji University School of Medicine, Daejeon, South Korea

**Keywords:** S100 protein, chronic eosinophilic leukemia, TLR4, FIP1L1-PDGFRα, imatinib resistance

## Abstract

S100A8 and S100A9 function as essential factors in inflammation and also exert antitumor or tumorigenic activity depending on the type of cancer. Chronic eosinophilic leukemia (CEL) is a rare hematological malignancy having elevated levels of eosinophils and characterized by the presence of the *FIP1L1-PDGFRA* fusion gene. In this study, we examined the pro-apoptotic mechanisms of S100A8 and S100A9 in FIP1L1-PDGFRα+ eosinophilic cells and hypereosinophilic patient cells. S100A8 and S100A9 induce apoptosis of the FIP1L1-PDGFRα+ EoL-1 cells via TLR4. The surface TLR4 expression increased after exposure to S100A8 and S100A9 although total TLR4 expression decreased. S100A8 and S100A9 suppressed the FIP1L1-PDGFRα-mediated signaling pathway by downregulating FIP1L1-PDGFRα mRNA and protein expression and triggered cell apoptosis by regulating caspase 9/3 pathway and Bcl family proteins. S100A8 and S100A9 also induced apoptosis of imatinib-resistant EoL-1 cells (EoL-1-IR). S100A8 and S100A9 blocked tumor progression of xenografted EoL-1 and EoL-1-IR cells in NOD-SCID mice and evoked apoptosis of eosinophils derived from hypereosinophilic syndrome as well as chronic eosinophilic leukemia. These findings may contribute to a progressive understanding of S100A8 and S100A9 in the pathogenic and therapeutic mechanism of hematological malignancy.

## Introduction

S100A8 and S100A9 are members of the S100 family proteins, having constitutive or inducible expressions in neutrophils, monocytes/macrophages, dendritic cells, and osteoclasts (1, 2). These proteins are included in the damage-associated molecular pattern (DAMP) and function as pro-inflammatory stimulators or enhancers through toll-like receptor 4 (TLR4). Recent research suggests that close association between cancer and inflammation is critical to unveil the pathogenic mechanism of tumors and development of therapeutic drugs for the disease (3, 4). S100 proteins are strongly related to antitumor effects as well as tumor progression and metastasis (5). S100A8 and S100A9 promote metastasis of breast carcinoma by forming a metastatic milieu and enhance apoptotic death in human head and neck squamous cell carcinomas and colorectal carcinoma (6–8).

Hypereosinophilia (HE) is a myeloproliferative disorder related to characteristically increased eosinophils in the bone marrow and peripheral blood, which infiltrate various organs and result in severe organ damage (9). The *FIP1(14.7K-interacting protein 1)-like-1–platelet-derived growth factor receptor-*α (*FIP1L1-PDGFRA*) fusion gene is a barometer distinguishing chronic eosinophilic leukemia (CEL) and other eosinophilic disorders, such as reactive eosinophilia (RE) and hypereosinophilic syndrome (HES) (10, 11). As per the WHO classification, chronic eosinophilic leukemia is also called myeloid/lymphoid neoplasm with eosinophilia and abnormalities of *PDGFRA, PDGFRB, FGFR1, or PCM1-JAK2* (1).

Recent studies on hematological malignancies have reported that the overexpression or downregulation of S100A8 and S100A9 is associated with acute myeloid leukemia (AML), chronic lymphoid leukemia (CLL), and myeloproliferative diseases (12–14) However, the function of S100A8 and S100A9 in chronic eosinophilic leukemia is not fully understood. This study was, therefore, undertaken to determine the antitumor effects of S100A8 and S100A9 on eosinophils of chronic eosinophilic leukemia.

## Materials and Methods

### Materials

RPMI 1640, penicillin and streptomycin solution, and fetal bovine serum (FBS) were purchased from Life Technologies Inc. TAK-242, an inhibitor of TLR4 (TLR4i), was obtained from Calbiochem (San Diego, CA, USA), respectively. Imatinib mesylate, a selective tyrosine kinase inhibitor, was obtained from Sigma. Anti-Bcl-2 antibodies (554160) were obtained from BD Biosciences. Anti-TLR4 (sc-10741), anti-ERK1/2 (sc-154), anti-β-actin (sc-47778), anti-pro-caspase 9 (sc-17784), anti-pro-caspase 3 (sc-7272), anti-phospho-AKT (sc-109903), anti-AKT (sc-8312), anti-PDGFRα (sc-398206), anti-S100A8 (sc-20174), anti-S100A9 (sc-20173), and anti-Ki-67 (sc-23900) antibodies were acquired from Santa Cruz Biotechnology (Santa Cruz). Z-DEVD-fmk (60332), anti-phospho-STAT3 (9131), anti-STAT3 (9139), anti-cleaved caspase 9 (9501), anti-cleaved caspase 3 (9664), anti-AIF (4642), anti-Bax (2772), anti-Mcl-1 (4572), anti-phospho-ERK1/2 (9101), anti-phospho-p38 MAPK (9211), anti-p38 MAPK (9212), anti-phospho-JNK (9251), anti-JNK (9252), anti-rabbit IgG-HRP (7074), and anti-mouse IgG-HRP (7076) antibodies were procured from Cell Signaling Technology. Anti-phospho-Bad (9296) and anti-Bad (9292) antibodies were purchased from New England Biolabs. Anti-phospho-PDGFRα antibodies (ab5460) for immunohistochemistry were obtained from Abcam.

### Production of Recombinant S100A8 and S100A9 Proteins

Briefly, cloned pET28a (+) vector (Novagen) bearing the cDNAs of human S100A8 and S100A9 was transformed to the expression vector *E. coli*, induced by 1 mM isopropyl β-D-thiogalactoside; the cells were subsequently cultured for an additional 4 and 16 h, respectively, at 37°C, followed by harvesting at 5,000 g for 10 min (15). Following cell lysis by sonication, the crude recombinant His-tag S100A8 and S100A9 were purified by the His-bind purification kit (Novagen) and FPLC. Purity of the protein was verified by SDS-PAGE. The endotoxin concentration was determined with toxin sensor chromogenic LAL endotoxin assay kit (GenScript, Piscataway, NJ, USA). Endotoxin was removed by ToxinEraser endotoxin removal kit (GenScript) as this step was needed. The purified proteins (endotoxin <0.1 EU/μg) were aliquoted and stored at −80°C until further experiments.

### Cell Culture

EoL-1 cells were obtained from the Ricken Cell Bank (Tsukuba). HL-60, K562, U937, and Jurkat cells were obtained from ATCC. EoL-1, HL-60, K562, U937, and Jurkat cells were maintained in RPMI 1640 supplemented with 10% or 20% heat-inactivated FBS, penicillin (100 U/mL), and streptomycin (100 μg/mL). The cultured cells were maintained at subconfluency in a 95% air/5% CO_2_ humidified atmosphere at 37°C.

### Production of Imatinib-Resistant EoL-1 Cells (EoL-1-IR)

EoL-1 cells were treated with 100 nM imatinib; dead cells were removed by low centrifugation when subculturing. Cells were continuously treated with imatinib for 6 months. The surviving cells were considered as EoL-1-IR as the survival rate was maintained more than 90% in the presence of 100 nM imatinib.

### Leukocyte Isolation

Human granulocytes and mononuclear leukocytes were isolated from heparinized peripheral blood of healthy persons and HE patients (including CEL, HES, reactive eosinophilia, and AML) using Ficoll–Hypaque gradient centrifugation. Isolated cells were subsequently subjected to hypotonic lysis followed by washing to remove erythrocytes. Eosinophils and neutrophils were separated from granulocytes using a CD16 microbead magnetic cell sorting kit (Miltenyi Biotec). Isolation of lymphocytes and monocytes was achieved by using the monocyte isolation kit II (Miltenyi Biotec). The cells were resuspended at a density of 3 x 10^6^ cells/ml in RPMI 1640 medium supplemented with 1% penicillin–streptomycin and 10% FBS. This method routinely yielded at least 95% cell purity as assessed by cell counting on cytospin after DiffQuick stain (Sysmex Corporation, Kobe, Japan).

### Normal Subjects and Patients

Patients with HE (including CEL, HES, reactive eosinophilia, and AML) and 10 normal subjects were recruited from Eulji University (EU14-33), Eulji Medical Center (2017-06-022), and Chungnam National University Hospital (2018-02-046) (Supplementary Table 1). The normal subjects did not indicate any history of disease and were asymptomatic.

### Detection of Apoptosis

Isolated cells were incubated with reagents of a Muse™ Annexin-V & Dead Cell kit (Merck KGaA) for detection of cell apoptosis. Apoptotic cells were analyzed using a Muse™ Cell Analyzer (Merck KGaA) and were determined as the percentage of cells showing annexin V+/PI- and annexin V+/PI+. For the morphological estimation of apoptosis, the cells were cytocentrifuged and stained with Wright staining solution.

### Evaluation of Surface TLR4 Expression

EoL-1 cells and EoL-1-IR cells were treated with S100A8 and S100A9, and the harvested cells were washed with PBS buffer. After blocking nonspecific binding with total mouse IgG, the cells were incubated at 4°C for 30 min with anti-TLR4 antibodies. Baseline staining was acquired by incubating with mouse IgG instead of anti-TLR4 antibodies. Following incubation and washings, the cells were incubated with FITC-conjugated goat anti-mouse IgG at 4°C for 30 min. Finally, the cells were washed and analyzed on a Guava easyCyte flow cytometer (Merck Millipore). Data was analyzed using the InCyte 3.1 software (Merck Millipore).

### Western Blotting

The cells were lysed in CETi lysis buffer (TransLab) for 30 min, after which the lysate was centrifuged and the supernatant was collected. The total protein concentration of the lysate was evaluated using a BCA protein assay kit (Thermo Scientific) standardized to BSA as per the manufacturer's instruction. Estimated cell lysates were then resolved by SDS-PAGE and transferred to nitrocellulose membranes. The blots were incubated with primary antibodies, and subsequently incubated with HRP-conjugated secondary antibodies, including goat anti-rabbit or rabbit anti-mouse antibodies. Finally, the blots were developed using an enhanced chemiluminescence detection system (Thermo Scientific) and visualized by Chemidoc (Bio-Rad).

### Real-Time PCR

RNAs from unstimulated and S100A8 or S100A9 treated EoL-1 cells were extracted using Trizol (Invitrogen) followed by column-purification with an RNeasy Mini Kit (Qiagen). The purified RNAs were treated with DNase I (New England Biolabs), and cDNA was synthesized using the iScript cDNA synthesis kit (Bio-Rad). Real-time PCR was performed using the iQ SYBR Green Supermix (Bio-Rad). PCR was performed using a CFX96 Real-Time System (Bio-Rad, Hercules, CA, USA). Sequences of primers for PDGFRα mRNA used were: forward 5′-GGG CAA ATG AGA ACA GCA ACA-3′ and reverse 5′-CTT CGT CTG AGA TAC TGG ATT CCT-3′.

### Tumor Xenograft Animal Experiment

Five-week-old female NOD-SCID (NOD.CB17-Prkdcscid/J) mice were purchased from Janvier Labs (Le Genest-Saint-Isle). For 1 week before initiating experiments, all animals were maintained in a specific pathogen-free facility of the Eulji University. The experimental mice were divided into six groups (*n* = 5 per a group): untreated, control, S100A8 treatment (0.5 and 1 mg doses), and S100A9 treatment (0.5 mg and 1 mg doses). The control, S100A8 treatment, and S1009 treatment groups were subcutaneously inoculated in the shoulders with EoL-1 cells (2 × 10^7^/100 μL) or EoL-1-IR cells (1 × 10^7^/100 μL) in RPMI 1640 medium. Treatment groups were subcutaneously administered 0.5 or 1 mg of S100A8 or S100A9 per group at 5 and 7 days after cell inoculation. The untreated group was injected with vehicle control (PBS). Tumor volumes were measured with caliper and calculated by applying the formula *a*^2^ × *b* × 0.4 (where “*a*” is the smallest diameter and “*b*” is the diameter perpendicular to “*a*”) as described in a previous paper (16). The body weight, behavior, and activity of mice were monitored for evaluating their general health. All animals were finally euthanized, and tumor xenografts were immediately harvested, weighed, and either lysed for Western blotting or fixed for immunohistochemistry.

### Histochemistry

After euthanizing the mice by CO_2_ asphyxiation, the tumor was harvested, fixed in formalin solution (Sigma), embedded in paraffin (Sigma), sectioned (5 μm thick slices), and stained with hematoxylin-eosin solution (Sigma). Immunohistochemical staining was performed on 3-μm-thick paraffin embedded sections. Briefly, the sections were placed on SuperfrostPlus microscope slides (Fisher Scientific). Vectastain elite ABC HRP kit (Vector Labs) was used as a 3,3′-diaminobenzidine (DAB) chromogen for detecting antibodies. Sections were deparaffinized by xylene. Solution of 0.1% trypsin was applied for 10 min for antigen retrieval, followed by treatment with 0.3% H_2_O_2_ in methanol for 30 min. Slides were blocked by blocking buffer and subsequently incubated with primary antibodies and secondary antibodies for 30 min each. After incubating with AB and DAB reagents, the specimens were counterstained with hematoxylin-eosin and examined under light microscopy (Leica Microsystems) for histological evaluation.

### Statistical Analysis

Values are expressed as the means ± standard deviation (*SD*). Intergroup differences were analyzed by applying the Student's *t*-test, and one-way ANOVA was applied for comparison of more than two groups, using the SPSS software, version 18.0 (SPSS). A *p* < 0.05 is considered to indicate statistical significance.

## Results

### S100A8 and S100A9 Induce EoL-1 Cell Apoptosis via TLR4

**Figure 1:**
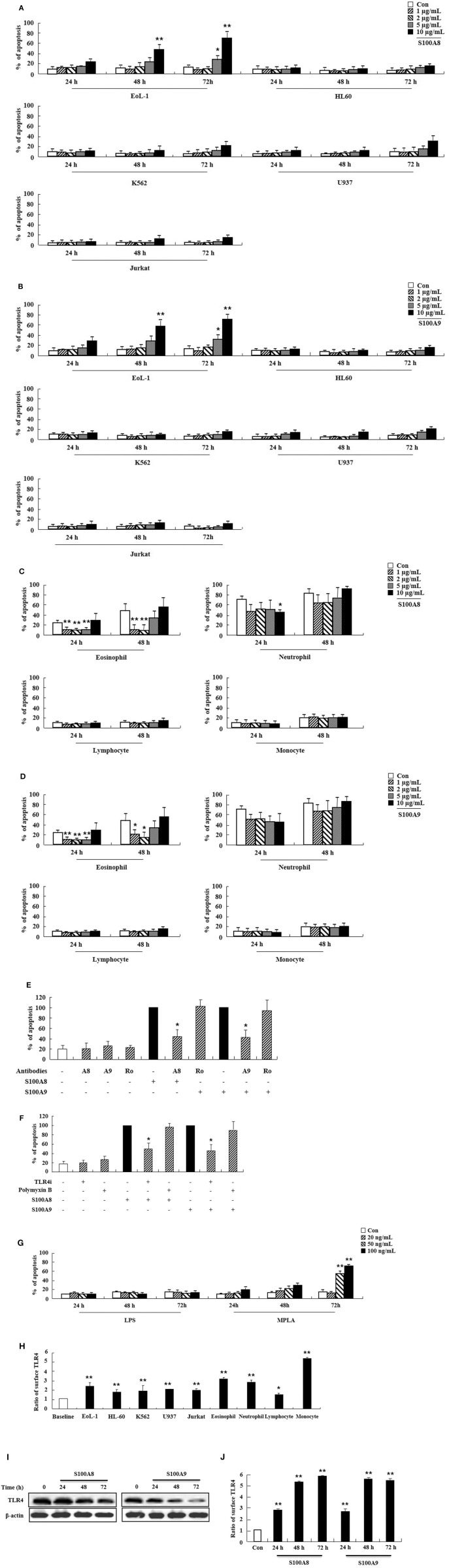
S100A8 and S100A9 induce EoL-1 cell apoptosis via TLR4. **(A–D)** EoL-1, HL-60, K562, U937, Jurkat cells **(A,B)**, eosinophils, neutrophils, lymphocytes, and monocytes isolated from normal subjects **(C,D)** were incubated for 24, 48, or 72 h in the absence (Con) or presence of indicated concentrations of S100A8 **(A,C)** and S100A9 **(B,D)** (3 < *n* < 5). **(E)** EoL-1 cells were pretreated with anti-S100A8 (A8), anti-S100A9 (A9), or rabbit control (Ro) antibodies (2 μg/mL) and subsequently incubated with S100A8 or S100A9 (10 μg/mL) (*n* = 4). **(F)** EoL-1 cells were pretreated with or without 1 μM TLR4 inhibitor (TLR4i) and polymyxin B (10 μg/mL) for 1 h, after which the cells were incubated for 72 h in the absence or presence of S100A8 and S100A9 (50 μg/mL) (3 < *n* < 5). Data are presented relative to the S100A8- or S100A9-treated group, which is set at 100% **(E,F)**. Data are expressed as the means ± *SD*. *p < 0.05 indicates a significant difference between the S100A8- or S100A9-treated group and stimulator-treated groups. **(G)** EoL-1 cells were treated with indicated concentrations of LPS and MPLA for 24, 48, and 72 h (*n* = 3). Apoptosis was analyzed by measuring the binding of annexin V-FITC and PI. **(H)** Flow cytometry was applied to determine TLR4 expression in EoL-1, HL-60, K562, U937, Jurkat cells, and normal eosinophils, neutrophils, lymphocytes, and monocytes without stimulators (3 < *n* < 5). Baseline fluorescence was obtained by incubating normal rabbit antibodies and was set at 1. **(I, J)** EoL-1 cells were incubated for 24, 48, and 72 h in the absence (Con) or presence of S100A8 and S100A9 (10 μg/mL) (*n* = 3). TLR4 expression was detected using Western blotting **(I)** and flow cytometry **(J)**. Data are expressed as the means ± *SD*. *p < 0.05 and **p < 0.01 indicate a significant difference between the control and stimulator-treated groups.

We first examined the effect of S100A8 and S100A9 on apoptosis of different leukemic cells such as HL-60, K562, U937, Jurkat, and EoL-1. S100A8 or S100A9 strongly induced the apoptosis of EoL-1 cells in a time- and dose-dependent manner but had little effect on other leukemia cells (Figures 1A,B). Combination of S100A8 and S100A9 treatment exerted no synergic effect on the apoptosis (Supplementary Figure 1). Contrarily, S100A8 and S100A9 showed anti-apoptotic effects on spontaneous apoptosis of the cells in normal eosinophils and neutrophils (Figures 1C,D). Low concentrations of S100A8 and S100A9 were more effective on apoptosis of normal eosinophils than higher concentrations. Survival of lymphocytes and monocytes were unaffected by S100A8 and S100A9 exposure. To confirm the apoptotic effects of S100A8 and S100A9, anti-S100A8 or anti-S100A9 antibodies were applied, which inhibited the pro-apoptotic effects by blocking the S100A8 and S100A9 (Figure 1E). For comparing the effects of S100A8 and S100A9 on leukemic cells and normal leukocytes, the eosinophils, neutrophils, lymphocytes, and monocytes were isolated from peripheral blood leukocytes. Because S100A8 or S100A9 transduces its signal via TLR4, alterations in the suppressive effects of S100A8 and S100A9 were investigated using TLR4i and assessing changes of TLR4 expression. As shown in Figure 1F, the TLR4 inhibitor blocks the apoptotic effect induced by S100A8 and S100A9. Polymyxin B did not alter the apoptosis induced by S100A8 and S100A9, indicating that the effects of recombinant S100A8 and S100A9 proteins are not affected by LPS contamination. To reinforce the association of TLR4 to the apoptotic effect, cells were treated with TLR4 agonists. Unexpectedly, the apoptosis of EoL-1 cells is not induced by lipopolysaccharide (LPS), but is induced by monophosphoryl Lipid A (MPLA) (Figure 1G, Supplementary Figure 2). We next examined the amount of TLR4 expression in EoL-1 cells. Our results showed strong TLR4 expression, similar to that observed in other leukemia cells, normal eosinophils, and neutrophils (Figure 1H). Surface TLR4 expression was strongly induced in a time-dependent manner although total TLR4 expression reduced after treatment with S100A8 and S100A9 (Figures 1I,J). Although there was a decrease in TLR4 expression during apoptosis, these results indicate that S100A8 and S100A9 promote apoptosis by increasing TLR4 expression after inducing apoptosis as a positive feedback loop.

### S100A8 and S100A9 Suppress the Downstream Signal of FIP1L1-PDGFRα by Decreasing Its Expression

**Figure 2:**
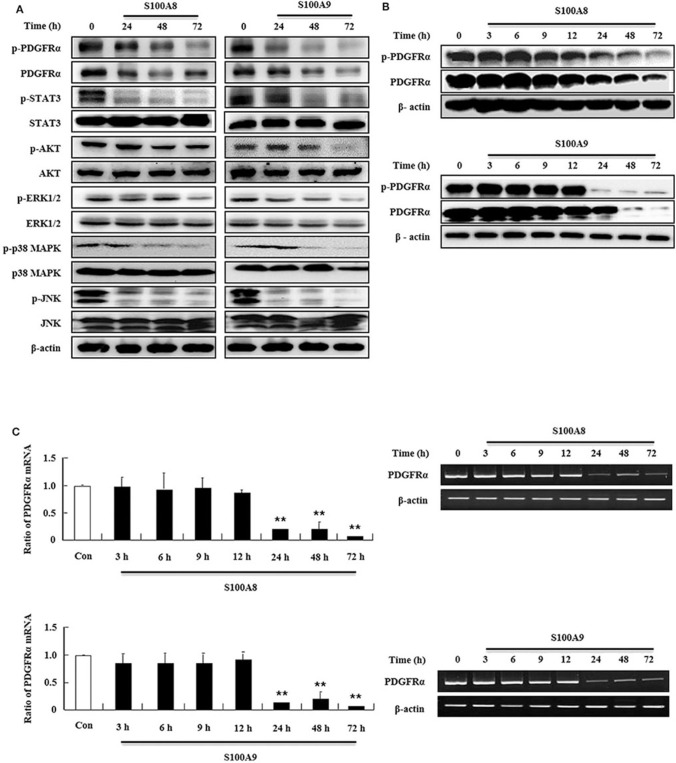
S100A8 and S100A9 suppress downstream signal of FIP1L1-PDGFRα by decreasing its expression. **(A)** EoL-1 cells were incubated with S100A8 and S100A9 (10 μg/mL) for 24, 48, and 72 h. After cell lysis, phosphorylation and non-phosphorylation of PDGFRα, STAT3, AKT, ERK1/2, p38 MAPK, and JNK were detected in the lysates by applying Western blotting. **(B,C)** EoL-1 cells were incubated with S100A8 and S100A9 (10 μg/mL) for the indicated time. After cell lysis, lysates were collected for detecting phosphorylation and non-phosphorylation of PDGFRα using Western blotting **(B)**. Total RNAs from EoL-1 cells were collected, and RT-PCR was conducted for detection of PDGFRα **(C)**. β-actin was used as an internal control. **p < 0.01 indicates a significant difference between the control and stimulator-treated groups.

In order to examine the apoptotic mechanism triggered by S100A8 and S100A9, we investigated the expression and activation of signal proteins involved in FIP1L1-PDGFRα. Constitutive phosphorylation of STAT3, AKT, and MAPKs was suppressed without alteration of their expression. Phosphorylation of PDGFRα decreased after treatment with S100A8 and S100A9, accompanied by downregulation of FIP1L1-PDGFRα expression (Figures 2A,B). Decrease of FIP1L1-PDGFRα protein expression was due to inhibition of the mRNA expression of FIP1L1-PDGFRα, which continued to decrease in the presence of S100A8 and S100A9 (Figure 2C). Thus, presence of S100A8 and S100A9 resulted in decreasing levels of FIP1L1-PDGFRα mRNA and protein, finally resulting in abolishment of the proliferation signal due to depleted FIP1L1-PDGFRα.

### S100A8 and S100A9 Induce Apoptosis via the Mitochondrial Apoptosis Pathway

**Figure 3:**
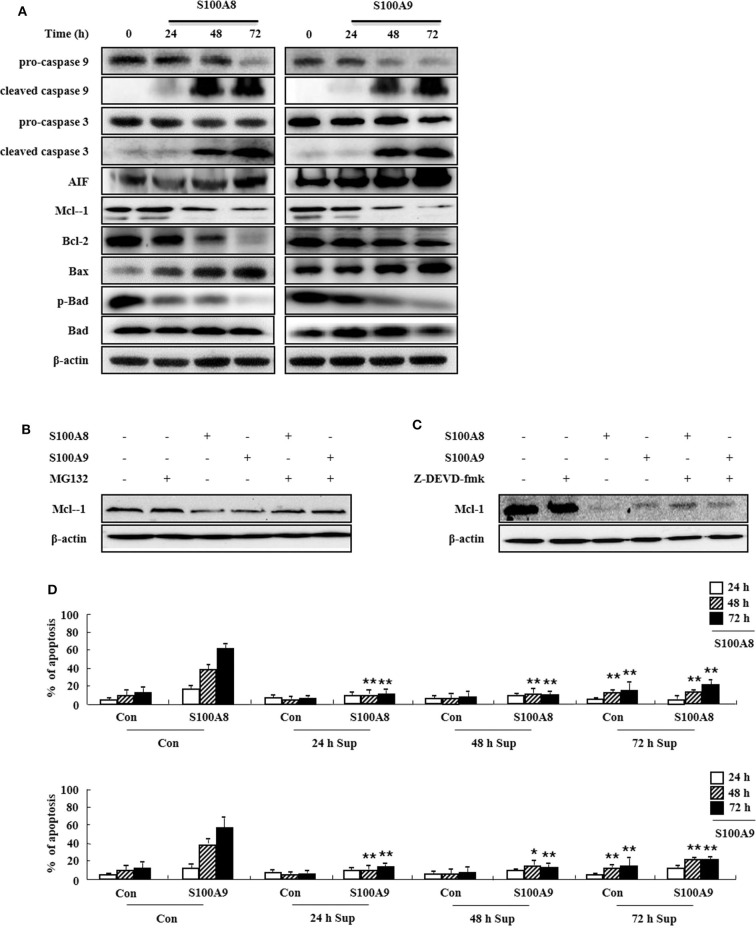
S100A8 and S100A9 are required for the mitochondrial apoptosis pathway. **(A)** EoL-1 cells were incubated with S100A8 and S100A9 (10 μg/mL) for 24, 48, and 72 h. After cell lysis, the pro-caspase 9, cleaved caspase 9, pro-caspase 3, caspase 3, cleaved caspase 3, AIF, Mcl-1, Bcl-2, Bax, phospho-Bad, and Bad in the lysates were detected by Western blotting. **(B,C)** EoL-1 cells were pretreated with or without 0.5 μM MG132 **(B)** and 10 μM z-DEVD-fmk **(C)** for 1 h, followed by incubation with S100A8 and S100A9 (10 μg/mL) for 48 h. Levels of Mcl-1 in the cell lysates were detected by Western blotting. β-actin was used as an internal control. **(D)** EoL-1 cells were incubated with S100A8 and S100A9 (10 μg/mL) for 24, 48, and 72 h, and supernatants were collected at appropriate time points. EoL-1 cells were incubated with S100A8 and S100A9 (10 μg/mL) for 24, 48, and 72 h in the absence or presence of the supernatant (*n* = 3). Apoptosis was analyzed by measuring the binding of annexin V-FITC and PI. *p < 0.05 and **p < 0.01 indicate a significant difference between the control and stimulator-treated groups.

To determine the cell apoptosis signaling pathway triggered by S100A8 and S100A9, we investigated alteration of mitochondria-involved signal proteins. As shown in Figure 3A, S100A8 and S100A9 activate the caspase 9/3 pathway by increasing levels of cleaved caspase 9 and caspase 3. AIF released from mitochondria are increased after S100A8 and S100A9 stimulation. It is well-documented that anti-apoptotic proteins (Mcl-1 and Bcl-2) and pro-apoptotic proteins (including Bax and Bad) are essential in mitochondrial damage. Exposure to S100A8 and S100A9 decreases expression of the Mcl-1 and Bcl-2/Bax ratio. Although Bad expression is not affected by S100A8 and S100A9, exposure resulted in decreased phosphorylation of Bad, which helps the Bad to translocate to the mitochondria. The decrease of Mcl-1 expression is suppressed by MG132 and Z-DEVD-fmk, indicating that Mcl degradation is regulated by proteasome and activated caspase 3 (Figures 3B,C). Because inflammatory cytokines induce an environment for tumor proliferation or suppression (17), we evaluated the effect of cytokines secreted from EoL-1 cells. Cell culture supernatant was collected from EoL-1 cells exposed to S100A8 and S100A9, which was subsequently used to stimulate cells in the absence or presence of S100A8 and S100A9. The supernatant blocked apoptosis induced by S100A8 and S100A9, indicating that secreted cytokines induce anti-apoptotic effects during apoptosis (Figure 3D). The supernatants also had little effect on apoptosis of normal leukocytes and other leukemia cells (Supplementary Figure 3). These results indicate that S100A8 and S100A9 induce contradictive pathways, i.e., both pro-apoptotic and anti-apoptotic mechanisms, and the intracellular apoptotic signal due to S100A8 and S100A9 is stronger than the survival signal induced by cytokines secreted by S100A8 and S100A9 stimulation. Because S100-mediated signaling is associated with the myeloid differentiation factor 88 (MyD88), Src family protein, PI3K, AKT, PKCs, MAPKs, and NF-κB, we examined the effects of inhibitors targeting these proteins, which are, in part, related to downstream signal protein of FIP1L1-PDGFRα as shown in Figure 2. We observed that inhibitors of MyD88, Src family protein, PI3K, AKT, PKCs, MAPKs, and NF-κB solely increased the apoptosis (Supplementary Figure 4).

### S100A8 and S100A9 Trigger Apoptotic Effects on EoL-1-IR Cells

**Figure 4:**
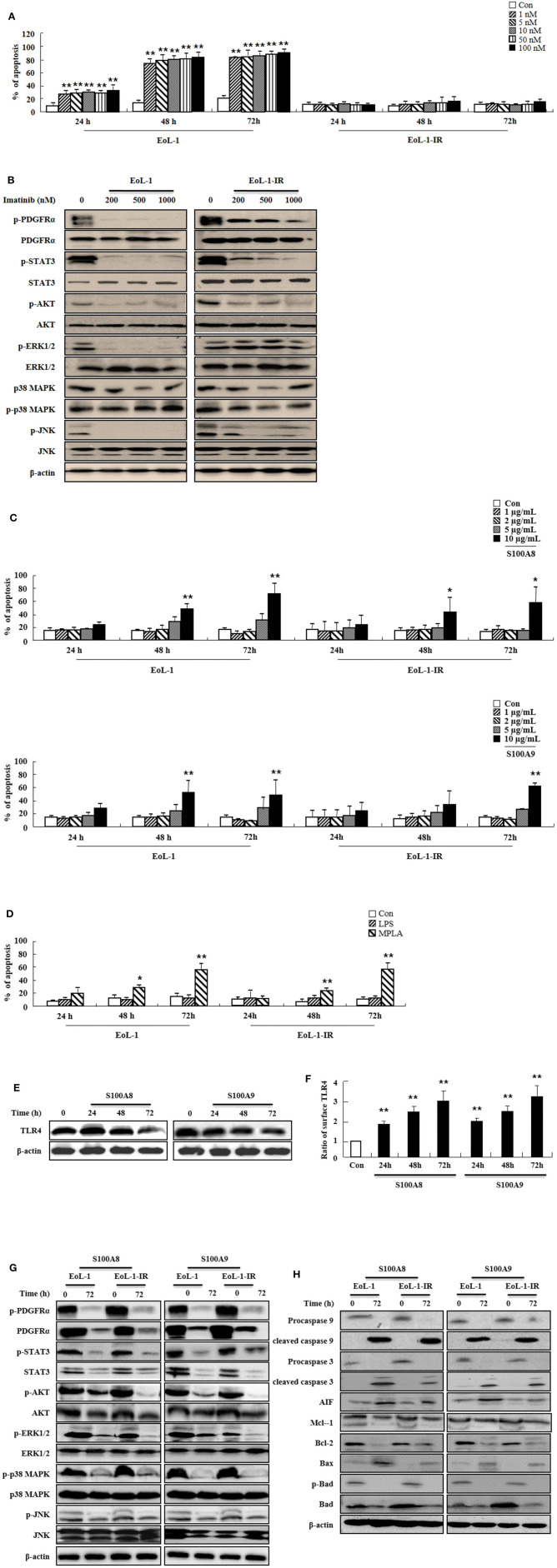
S100A8 and S100A9 trigger apoptotic effects on EoL-1-IR cells. **(A)** EoL-1 and EoL-1-IR cells were incubated for 24, 48, and 72 h in the absence (Con) or presence of imatinib at the indicated concentration. Apoptosis was analyzed by measuring the binding of annexin V-FITC and PI. **(B)** EoL-1 and EoL-1-1R cells were incubated for 72 h in the absence or presence of imatinib at the indicated concentration. Following cell lysis, phosphorylation and non-phosphorylation of PDGFRα, STAT3, AKT, ERK1/2, p38 MAPK, and JNK in the lysates were detected by Western blotting. **(C,D)** EoL-1 and EoL-1-IR cells were incubated for 24, 48, and 72 h in the absence (Con) or presence of indicated concentrations of S100A8, S100A9 **(C)**, LPS (100 ng/mL) and MPLA (100 ng/mL) **(D)** (*n* = 3). Apoptosis was analyzed by measuring the binding of annexin V-FITC and PI. **(E,F)** EoL-1-IR cells were incubated for 24, 48, and 72 h in the absence (Con) or presence of S100A8 and S100A9 (10 μg/mL), and TLR4 expression was detected using Western blotting **(E)** and flow cytometry **(F)**. Data are expressed as the means ± *SD*. *p < 0.05 and **p < 0.01 indicate a significant difference between the control and stimulator-treated groups. **(G,H)** EoL-1 cells were incubated with S100A8 and S100A9 (10 μg/mL) for 72 h. After cell lysis, the indicated protein expression in the lysates was detected by Western blotting. β-actin was used as an internal control.

Because leukemia patients develop resistance to imatinib after prolonged treatment and show severe adverse clinical implications, we investigated the efficacy of S100A8 and S100A9 against imatinib resistance. The EoL-1-IR cell line was established after prolonged stimulation with 100 nM imatinib (6 months). As shown in Figure 4A, our established EoL-1-IR cells are resistant to concentrations ranging from 1 nM to 100 nM of imatinib in contrast to the imatinib-sensitive EoL-1 cells (Figure 4A). In the presence of >200 nM imatinib, activation of FIP1L1-PDGFRα-mediated signal proteins was decreased due to reduced phosphorylation of PDGFRα in both EoL-1 and EoL-1-IR cells without any alteration in the PDGFRα expression (Figure 4B). Although EoL-1 and EoL-1-IR cells are sensitive at higher concentrations of imatinib, we found that EoL-1-IR cells are comparatively more resistant than the EoL-1 cells. Moreover, S100A8 and S100A9 induce remarkable increase in apoptosis in a time- and dose-dependent manner regardless of the imatinib-resistance (Figure 4C). MPLA also induces apoptotic death, but LPS had no effect on apoptosis (Figure 4D). EoL-1-1R cells also show decreased total expression of TLR4, similar to the change of TLR4 expression in EoL-1 cells; however, the EoL-1-1R cells show a time-dependent increase in the surface expression of TLR4 (Figures 1G,H, 4E,F). We further explored how S100A8 and S100A9 induce apoptosis in EoL-1-1R as compared to EoL-1 cells. S100A8 and S100A9 elicit two distinct apoptotic pathways in EoL-1-1R, similar to EoL-1 cells. S100A8 and S100A9 potently reduce the downstream signal transduction of FIP1L1-PDGFRα by downregulating protein expression of PDGFRα via TLR4, which is different from the suppressive mechanism due to imatinib (Figures 4B,G). In addition, S100A8 and S100A9 direct their pro-apoptotic effects through mitochondrial-associated apoptosis mechanism (Figure 4H). After exposure to S100A8 and S100A9, caspase 9 and caspase 3 are cleaved, subsequent to their activation. The expression of Mcl-1 and Bcl-2 and phosphorylation of Bad is diminished, and Bax expression is increased. Alterations of basal signal proteins seen in these results were not observed in the EoL-1-1R cells, when compared to EoL-1 cells (Figures 4B,G,H).

### S100A8 and S100A9 Suppress Tumorigenesis Induced by Xenograft of EoL-1 and EoL-1-IR in NOD-SCID Mice

**Figure 5:**
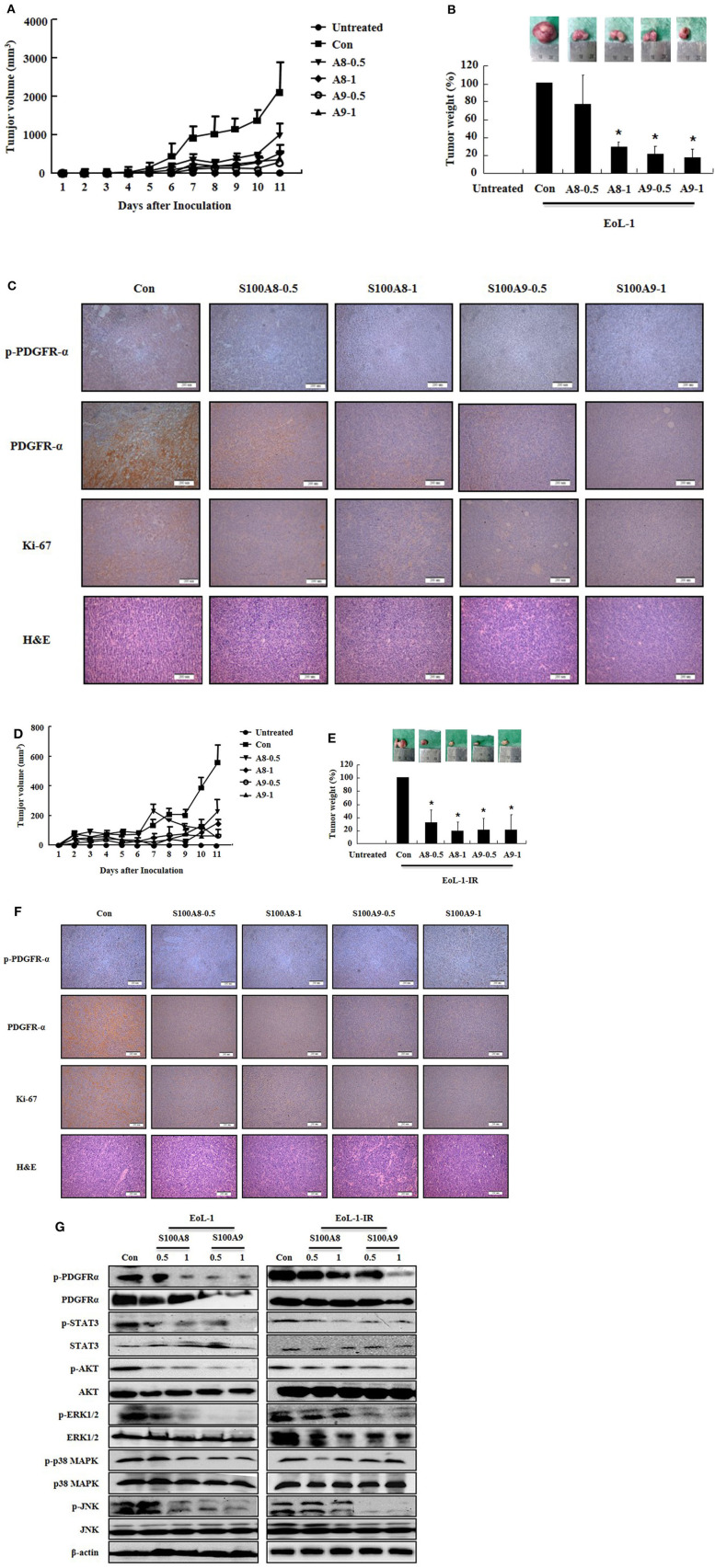
S100A8 and S100A9 suppress tumorigenesis induced by xenograft of EoL-1 and EoL-1-1R in NOD-SCID mice. **(A–F)** Five-week-old female NOD-SCID (NOD.CB17-Prkdcscid/J) mice were divided into six groups (*n* = 5 per a group) as described in the materials and methods: untreated, control, S100A8 treatment (0.5 and 1 mg doses), and S100A9 treatment (0.5 and 1 mg doses). The control, S100A8 treatment, and S1009 treatment groups were subcutaneously inoculated in the shoulder with EoL-1 cells (2 × 10^7^/100 μL) **(A–C)** or EoL-1-IR cells (1 × 10^7^/100 μL) **(D–F)**. Treatment groups were subsequently administered subcutaneous doses of 0.5 or 1 mg S100A8 or S100A9 at days 5 and 7 after cell inoculation. The untreated group was injected with vehicle (PBS). Tumor volume (day 1–11) and tumor weight (day 11) were measured **(A,B,D,E)**. Tumor mass was examined for detecting phospho-PDGFRα, PDGFRα, and Ki-67, applying immunohistochemistry and hematoxylin and eosin stain (Scale bar: 200 μm) **(C,F)**, or subjected to Western blotting to determine phosphorylation and nonphosphorylation of PDGFRα, STAT3, AKT, ERK1/2, p38 MAPK, and JNK **(G)**. β-actin was used as the internal control. *p < 0.05 indicates a significant difference between the control and stimulator-treated groups.

After identifying the *in vitro* effects of S100A8 and S100A9 in cells, we next undertook to confirm the *in vivo* effects of S100A8 and S100A9 using immune-deficient mice. After subcutaneous inoculation of EoL-1 cells (2 × 10^7^/100 μL) or EoL-1R cells (1 × 10^7^/100 μL), we evaluated the tumor volume and weight and the alteration of PDGFRα activation by immunohistochemistry and Western blotting. The xenografted cells multiplied in a time-dependent manner, and tumor volume and weight were observed to decrease after administration of S100A8 and S100A9 (Figures 5A,C). Although tumor sizes differed in EoL-1 and EoL-1-1R cells due to initial inoculation of different cell numbers, the effects of S100A8 and S100A9 did not show specific differences. Results from immunohistochemistry and Western blotting confirmed that exposure to S100A8 and S100A9 reduces the phosphorylation of PDGFRα by decreasing the PDGFRα expression (Figures 5C,F,G). The treated group also shows diminished activation of Stat 3, Stat 5, AKT, ERK, p38 MAPK, and JNK (Figure 5G). In addition, Ki-67, a proliferation marker, is potently decreased after S100A8 and S100A9 administration (Figures 5C,F).

### S100A8 and S100A9 Elicit Eosinophil Apoptosis of HES Patients as Well as CEL Patients

**Figure 6:**
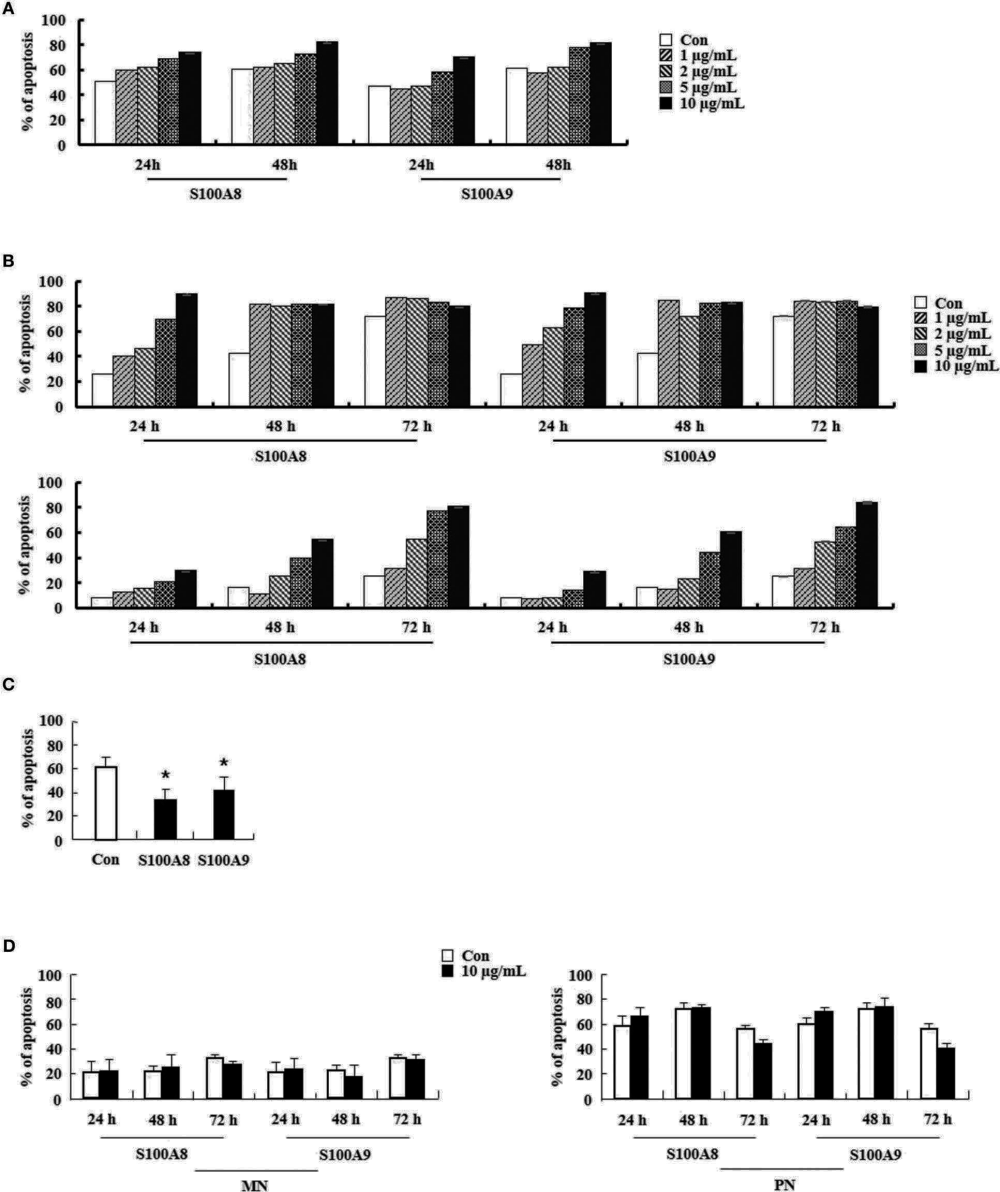
S100A8 and S100A9 elicit apoptosis of eosinophils isolated from CEL and HES patients. **(A–D)** Eosinophils were isolated from CEL (*n* = 1) **(A)**, HES (*n* = 2) **(B)**, and reactive eosinophilia subjects (*n* = 5) **(C)**, and polymorphonuclear cells and mononuclear cells were separated from AML subjects (*n* = 5) **(D)**. Eosinophils from CEL, HES, and AML were incubated for 24, 48, or 72 h, and the cells from reactive eosinopohilia were incubated for 48 h with the indicated concentrations of S100A8 and S100A9. Apoptosis was analyzed by measuring the binding of annexin V-FITC and PI. Data are expressed as the means ± *SD*. *p < 0.05 indicates a significant difference between the control and stimulator-treated groups.

We examined the effects of S100A8 and S100A9 in HE subjects with CEL, HES, and reactive eosinophilia-induced allergies. Apoptosis of eosinophils isolated from FIP1L1-PDGFRα-positive CEL is sensitive to stimulation with S100A8 and S100A9, and FIP1L1-PDGFRα-negative HES eosinophils also undergo apoptosis after exposure to S100A8 and S100A9 (Figures 6A,B). As shown in Figures 6C,D, eosinophil apoptosis of reactive eosinophilia is inhibited by S100A8 and S100A9 treatment. However, S100A8 and S100A9 have no effect on apoptosis of myeloid leukemia cells. Collectively, our *in vivo* findings demonstrate that S100A8 and S100A9 practically regulate the apoptotic mechanisms of eosinophilic abnormalities such as CEL and HES.

## Discussion

**Figure 7:**
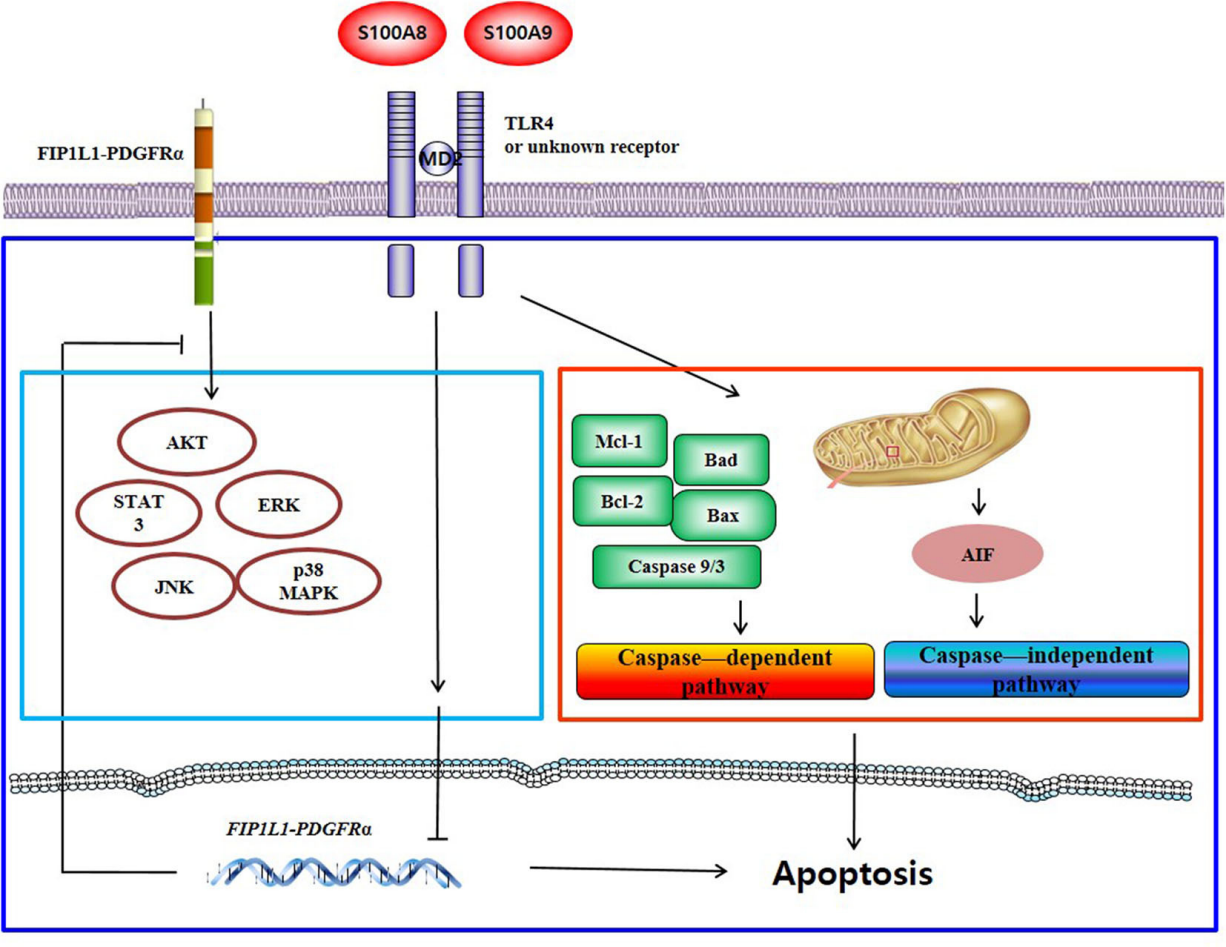
Proposed apoptotic mechanism due to S100A8 and S100A9 in CEL eosinophils. S100A8 and S100A9 trigger apoptosis of chronic eosinophilic leukemia by suppressing FIP1L1-PDGFRα+-mediated proliferation by downregulating the mRNA and protein expression and by inducing mitochondrial-associated apoptosis via TLR4.

S100A8 and S100A9 induce apoptosis via TLR4 by two mechanisms: one pathway decreases the expression of mRNA and proteins of FIP1L1-PDGFRα and its downstream pathway, and another pathway activates or inhibits the expression or activity of mitochondria-associated proteins such as caspase 9, caspase 3, Bcl-2, Bax, Mcl-1, Bad, and AIF. Putative mechanisms of S100A8 and S100A9 are presented in Figure 7.

Although TLR4 expression in EoL-1 cells is similar to that in other leukemia cells, EoL-1 cells undergo apoptosis subsequent to S100A8 and S100A9 treatment (Figure 1). Conversely, TLR4 signaling induced by S100A8 and S100A9 mediates the suppression of apoptosis in normal eosinophils and neutrophils. The difference between EoL-1 cells and normal cells is related to the existence of FIP1L1-PDGFRα+. Mutated receptors affect the intracellular mechanism and alter the response to extracellular stimulators as well as proliferation. This is reported in a study that states the overexpression of S100A9 appears in estrogen receptor α and progesterone receptor-negative breast cancer and inflammatory cytokines due to S100A9 and is dependent on TLR4 and EGFR (18). It is a popular concept that TLR4 is an essential receptor of S100A8 or S100A9 (19). Yang et al. suggest the importance of RAGE, another S100A8- or S100A9-sensing receptor, and the S100A8 promotion of autophagy via RAGE, which is not associated with TLR4 in leukemia cells (20). S100A9 regulates immune suppression via RAGE in colorectal cancer (21). In lung injuries, S100A8 transduces its signal via TLR4 and is not mediated by RAGE, which sometimes functions as an inhibitory receptor of AKT proliferation signaling in myeloproliferative neoplasms (14, 22). We, therefore, investigated the RAGE expression in cells. Alteration of RAGE after exposure to S100A9 and S100A9 is similar to the patterns observed for TLR4 expression (Supplementary Figure 5) (Figures 1I,J, 4E,F). Even though this is not direct evidence, it is desirable for investigating the association of RAGE in apoptosis induced by S100A8 and S100A9. In addition, S100A9 provokes differentiation of AML through TLR4, and S100A8 inhibits the differentiation by unknown mechanisms (23). This study revealed no differences between functional mechanisms of S100A8 and S100A9.

The expression of FIP1L1-PDGFRα is not altered by tyrosine kinase inhibitors, such as imatinib and ponatinib. However, SNS-032 [an inhibitor of cyclin-dependent kinase (CDK)7/9)] and triptolide (component of a plant extract) suppresses the expression of FIP1L1-PDGFRα by diminishing phosphorylation of RNA polymerase II (24, 25). It takes a longer duration to diminish PDGFRα after exposure to S100A8 and S100A9 as compared to the former inhibitor because S100A8 and S100A9 are extracellular ligands that transduce their signal via TLR4. Although the accurate mechanism of FIP1L1-PDGFRα gene expression is unknown, numerous transcription regulators (including transcription factor) may be modulated by TLR4 or certain signal pathways triggered by S100A8 and S100A9 (26). In addition, we observed the role of TLR4 in the anti-apoptotic mechanism by applying LPS and MPLA. Our results reveal that MPLA induces apoptosis in contrast to no effect observed by LPS stimulation (Figures 1G, 4D). The agonist-activated TLR4 transfers its signal via the myeloid differentiation factor 88 (MyD88)-dependent and MyD88-independent pathways, and the toll-interleukin 1 receptor domain-containing adapter inducing interferon-beta (TRIFF)–involved pathway (27, 28). Although LPS activates both MyD88 and TRIFF, several reports demonstrate that the action of MPLA is more dependent on TRIFF than LPS, which induces strong activation of the MyD88 pathway (29, 30). TLR4 is associated with cell death by TRIFF activation (31). The inhibitory peptide of MyD88 exerts no inhibition on the outcomes of S100A8 and S100A9 (Supplementary Figure 4). Collectively, these results indicate that the effect of S100A8 and S100A9 as observed in this study may rely on the TRIFF pathway. Conversely, MPLA is mediated more by MyD88 than TRIFF, and the S100 proteins are not involved in TRIFF pathway (31, 32). The effects of S100A8 and S100A9 on leukemia are induced by more complex mechanisms beyond our understanding. Further studies are required to elucidate the exact mechanisms involved in inducing the apoptotic effect of S100A8 and S100A9, including relation of TLR4, RAGE, FIP1L1-PDGFRα, and other novel proteins.

Regulation of FIP1L1-PDGFRα expression is essential for the action of S100A8 and S100A9 via TLR4 in EoL-1 and CEL patient cells. However, HES eosinophils without FIP1L1-PDGFRα expression are sensitive to S100A8 and S100A9, which results in induction of dramatic apoptosis (Figure 6B). Eosinophils are included in polymorphonuclear cells (PNCs). However, apoptosis of PNCs of HES is not affected by S100A8 and S100A9; rather, the mononuclear cells (MNCs) are sensitive to S100A8 and S100A9 exposure as compared to AML cells (Figure 6D) (data not shown). Therefore, we isolated eosinophils from PNCs by negative selection, which resulted in our finding that apoptosis of HES eosinophils are also induced by S100A8 and S100A9. Eosinophil purification from PNCs may be critical for unveiling the exact reaction of eosinophils. Furthermore, S100A8 and S100A9-mediated apoptosis in MNCs may indicate that eosinophils have heterogeneous characteristics. Considering the above data, numerous approaches were examined to elucidate the roles of S100A8 and S100A9. Despite limitation of samples owing to rarity of the diseases, HE (including HES as well as FIP1L1-PDGFRα+ CEL) needs to be investigated altogether, and S100A8 and S100A9 may have common and original apoptotic mechanisms, depending on diseases.

After treatment with tyrosine kinase inhibitors, such as imatinib, some patients with leukemia develop resistance to the treatment by mutation of target abnormal genes, resulting in the development of second-/third-generation drugs, such as dasatinib and ponatinib (33). We established the EoL-1-IR cell line, and each cloned EoL-1-IR cell was pooled in our experiments because acquired imatinib resistance is caused by multiple mechanisms (34–36). A recent paper recommends that maintenance therapy of imatinib is more essential than altered treatment with other generation drugs; however, we need to prepare beforehand for overcoming imatinib resistance (36). Overall, S100A8 and S100A9 induce apoptosis of EoL-1-IR cells by the same apoptotic mechanism in EoL-1 cells, as observed both *in vitro* and *in vivo* (Figures 4, 5). The action of S100A8 and S100A9 can, therefore, be useful in therapies for imatinib-sensitive or imatinib-resistant CEL. Because our results are unable to completely explain the usefulness of S100A8 and S100A9, it is imperative to unveil the drug-resistance mechanism for personal therapy and develop novel resistant drugs (including peptides and chemical compounds) based on TLR4 agonists, such as S100A8 and S100A9. Therapeutic drugs that target TLR4 and S100A8 or S100A9 have been examined, and potential candidate agents have been discovered (37–40). New materials based on our research can proceed in the quest for activators that induce TLR4-mediated apoptotic signal transduction and inhibitors of transcription-associated components that control transcription of the FIP1L1-PDGFRα+ gene. As shown in Figure 6, S100A8 and S100A9 promote the apoptosis of eosinophils from HES subjects as well as CEL patients, which indicates that both proteins possess therapeutic potential for both diseases but may also induce unwanted and unrecognized side effects, including inflammation (Figures 3D, 6).

In conclusion, this study demonstrates that S100A8 and S100A9 trigger apoptosis of chronic eosinophilic leukemia by induction of intrinsic apoptosis and suppression of FIP1L1-PDGFRα+-mediated proliferation. Therefore, we believe that our data will help to shed light on elucidating the inflammatory molecules available for therapeutic development of HE.

## Data Availability Statement

The datasets generated for this study are available on request to the corresponding author.

## Ethics Statement

The studies involving human participants were reviewed and approved by Human study was approved by the Institutional Review Board of Eulji University for normal volunteers and the Institutional Review Board of Chungnam National University Hospital and Eulji Medical Center for patients with CEL, HES, and reactive eosinophilia. All participants in this study gave their written informed consent. The patients/participants provided their written informed consent to participate in this study. The animal study was reviewed and approved by All animal studies were conducted with the approval of the Eulji University Institutional Animal Care and Use Committee.

## Author Contributions

J-SL designed the research study, wrote the manuscript, and analyzed the data. NL, AK, S-JY, and AN performed the experiments and analyzed the data. I-CS and S-JG provided the fresh clinical samples and managed the patients. MH, GK, and PS performed the experiments. M-SL and K-HS performed the experiments and discussed the data. IK conceived the concept, designed the experiments, and reviewed the paper. All authors read and approved the final manuscript.

## Conflict of Interest

The authors declare that the research was conducted in the absence of any commercial or financial relationships that could be construed as a potential conflict of interest.
