# Supplementary material for: S100A8 and S100A9 Promote Apoptosis of Chronic Eosinophilic Leukemia Cells
Source: Front Immunol. 2020 Aug 6;11:1258. doi: 10.3389/fimmu.2020.01258 (PMC7438788; doi:10.3389/fimmu.2020.01258)
Supplement: Supplementary file 1 [file Table_1.DOCX]

**Supplementary Table 1. Patient characteristics**

|  | **CEL** | **HES** | **RE** | **AML** |
| --- | --- | --- | --- | --- |
| **Number of subjects (female/male)** | 1 (1/0) | 2 (1/1) | 5(2/3) | 5 (4/1) |
| **Age (years)** | 64 | 56 ± 3 (53~59) | 37.8 ± 8.2 (24~45) | 58 .7 ± 8.6 (51~68) |
| **F/P fusion** | Positive | Negative | Negative | Negative |
| **Eosinophil (%)** | 20.6 | 14.7 ± 3.1 (12.5~16.9) | 6.1 ± 5.3 (1.7~12.3) | 0.5 ± 0.5 (0~1) |

CEL: chronic eosinophilic leukemia

HES: hypereosinophilic syndrome

RE: reactive eosinophilia

AML: acute myeloid leukemia

F/P: *FIP1L1-PDGFRA* fusion

Data are expressed as the means ± SD (the lowest value ~ the highest value)
