# Supplementary material for: S100A8 and S100A9 Promote Apoptosis of Chronic Eosinophilic Leukemia Cells
Source: Front Immunol. 2020 Aug 6;11:1258. doi: 10.3389/fimmu.2020.01258 (PMC7438788; doi:10.3389/fimmu.2020.01258)
Supplement: Supplementary file 2 [file Table_2.DOCX]

**Supplementary figure legends**

**Supplementary Figure 1.** **S100A8 and S100A9 induce EoL-1 cell apoptosis.** EoL-1, HL-60, K562, U937, and Jurkat cells were incubated for 24 h, 48 h or 72 h in the absence (Con) or presence of indicated concentrations of S100A8 and S100A9 (3< n<5). Apoptosis was analyzed by measuring the binding of annexin V-FITC and PI. Data are expressed as the means ± SD. **p* < 0.01 indicates a significant difference between the control and stimulator-treated groups.

**Supplementary Figure 2. LPS and MPLA have no effect on survival of other leukemia cells.** HL-60, K562, U937, and Jurkat cells were treated with indicated concentrations of LPS and MPLA for 24 h, 48 h and 72 h (n=3). Apoptosis was analyzed by measuring the binding of annexin V-FITC and PI. Data are expressed as the means ± SD.

**Supplementary Figure 3. The supernatant treated with S100A8 and S100A9 has no effect on survival of other leukemia cells and normal leukocytes.** (A, B) HL-60, K562, U937, Jurkat cells (A), eosinophils, neutrophils, lymphocytes and monocytes isolated from normal subjects (B) were incubated for 24 h, 48 h or 72 h in the absence (Con) or presence of indicated concentrations of S100A8 and S100A9, and supernatants were collected at appropriate time points. EoL-1 cells were incubated with S100A8 and S100A9 (10 µg/mL) for 24 h, 48 h, and 72 h in the absence or presence of the supernatant (n=3). Apoptosis was analyzed by measuring the binding of annexin V-FITC and PI.

**Supplementary Figure 4.** **EoL-1 cell apoptosis due to S100A8 and S100A9 is not affected by a MyD88 inhibitor.** EoL-1 cells were pre-treated with 100 µM MyD88-blocking peptide, 10 µM PP2, 10 µM LY294002 (LY), 10 µM AKT inhibitor (AKTi), 5 µM rottlerin (RT), 100 nM Ro-31-8420 (RO), 10 µM SP600125 (SP), 10 µM PD98059 (PD), 10 µM SB202190 (SB) and 2 µM BAY-11-7085 (BAY), and subsequently incubated with S100A8 or S100A9 (10 µg/mL) (n=3). Apoptosis was analyzed by measuring the binding of annexin V-FITC and PI. Data are presented relative to the S100A8 or S100A9–treated group, which is set at 100%, and are expressed as the means ± SD.

**Supplementary Figure 5.** **EoL-1 cell apoptosis due to S100A8 and S100A9 is not affected by a MyD88 inhibitor.** (A) Flow cytometry was applied to determine RAGE expression in EoL-1, HL-60, K562, U937, Jurkat cells, and normal eosinophils, neutrophils, lymphocytes and monocytes without stimulators (n=3). (B, C) EoL-1 (B) and EoL-1-IR cells (C) were incubated for 24 h, 48 h, and 72 h in the absence (Con) or presence of S100A8 and S100A9 (10 µg/mL) (n=3). RAGE expression was detected using Western blotting (left panel) and flow cytometry (right panel). Data are expressed as the means ± SD. ***p* < 0.05 and ***p* < 0.01 indicate a significant difference between the control and stimulator-treated groups.
