# Supplementary material for: S100A8 and S100A9 Promote Apoptosis of Chronic Eosinophilic Leukemia Cells
Source: Front Immunol. 2020 Aug 6;11:1258. doi: 10.3389/fimmu.2020.01258 (PMC7438788; doi:10.3389/fimmu.2020.01258)
Supplement: Supplementary file 3 [file Presentation_1.PPT]

## Slide 1
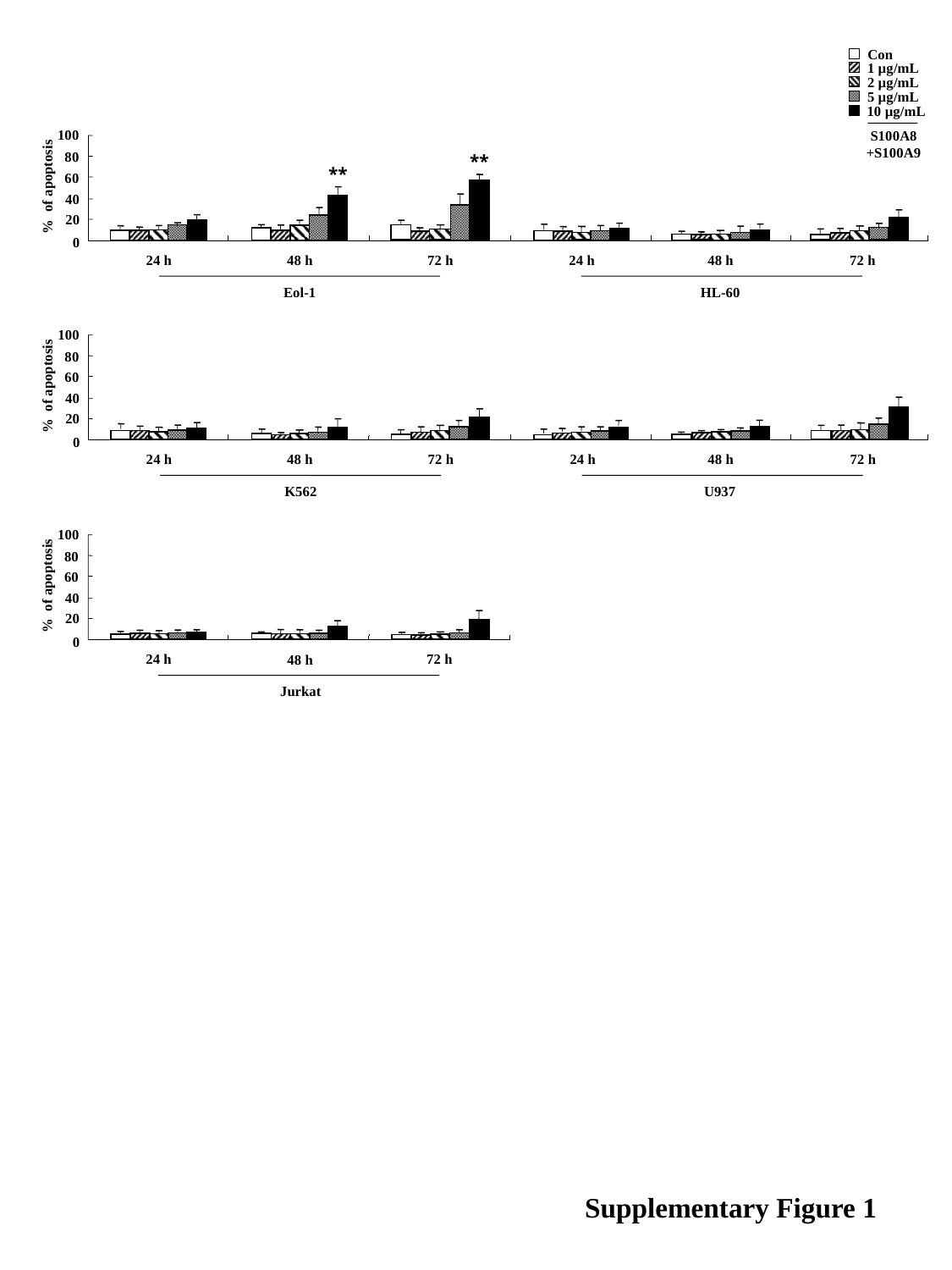

Con
1 µg/mL
2 µg/mL
5 µg/mL
10 µg/mL
S100A8
+S100A9
100
80
% of apoptosis
60
40
20
0
**
**
24 h
48 h
72 h
24 h
48 h
72 h
HL-60
Eol-1
100
80
% of apoptosis
60
40
20
0
24 h
48 h
72 h
24 h
48 h
72 h
U937
K562
100
80
% of apoptosis
60
40
20
0
24 h
72 h
48 h
Jurkat
Supplementary Figure 1

## Slide 2
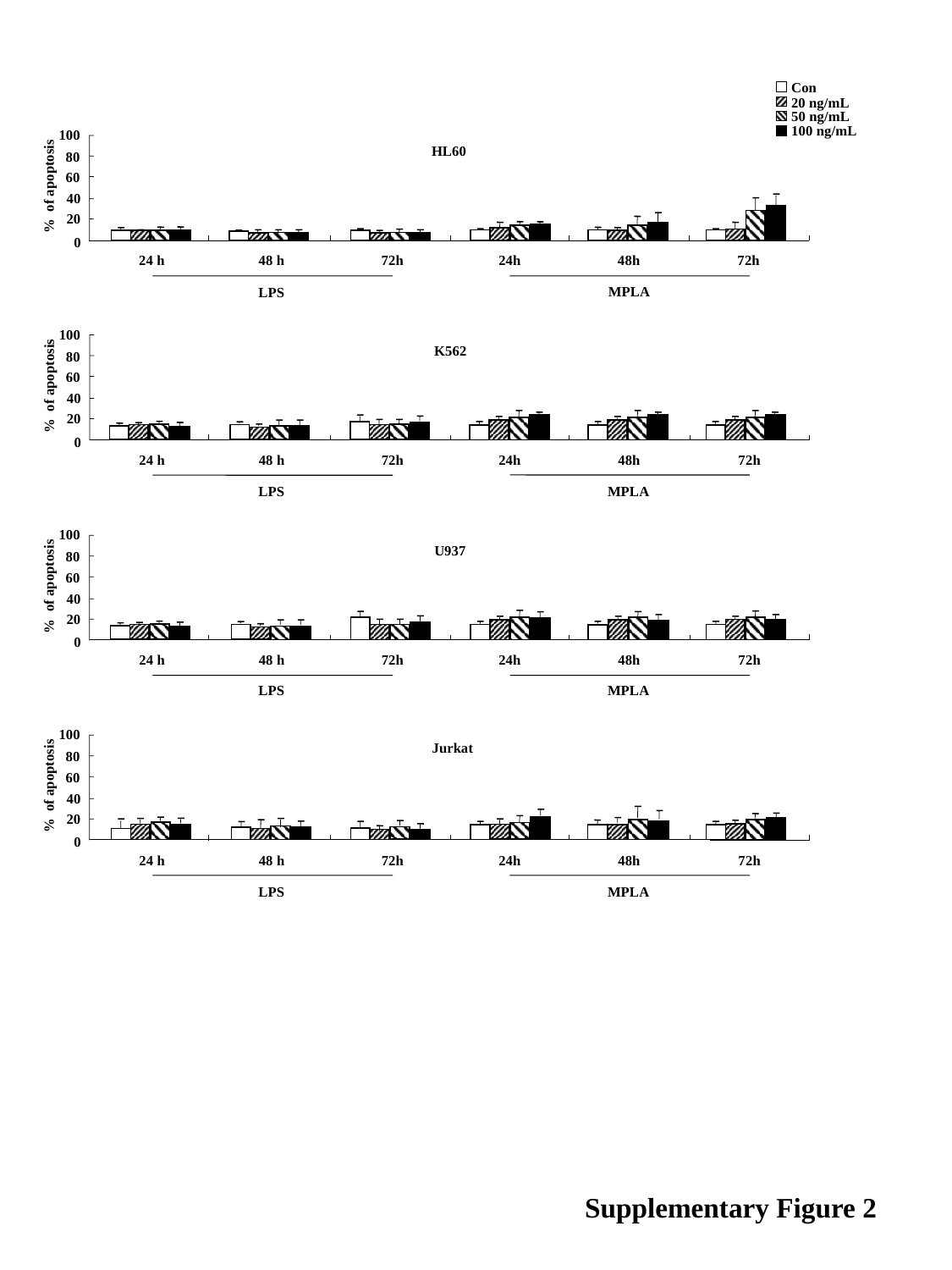

Con
20 ng/mL
50 ng/mL
100 ng/mL
100
HL60
80
% of apoptosis
60
40
20
0
24 h
48 h
72h
24h
48h
72h
MPLA
LPS
100
K562
80
% of apoptosis
60
40
20
0
24 h
48 h
72h
24h
48h
72h
LPS
MPLA
100
U937
80
% of apoptosis
60
40
20
0
24 h
48 h
72h
24h
48h
72h
MPLA
LPS
100
Jurkat
80
% of apoptosis
60
40
20
0
24 h
48 h
72h
24h
48h
72h
LPS
MPLA
Supplementary Figure 2

## Slide 3
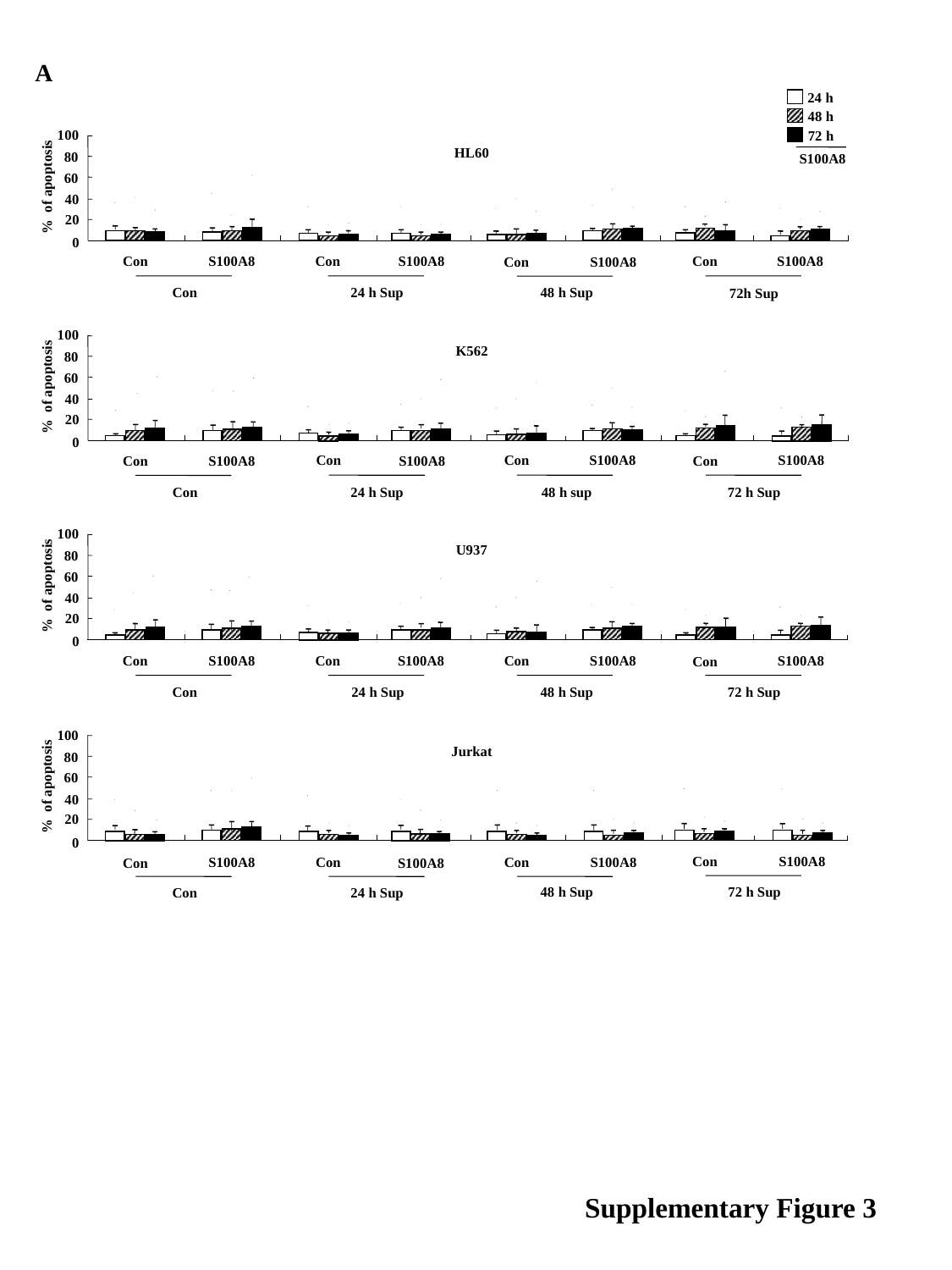

A
24 h
48 h
72 h
100
HL60
S100A8
80
% of apoptosis
60
40
20
0
S100A8
Con
S100A8
Con
S100A8
Con
Con
S100A8
Con
24 h Sup
48 h Sup
72h Sup
100
K562
80
% of apoptosis
60
40
20
0
Con
Con
S100A8
S100A8
S100A8
S100A8
Con
Con
Con
24 h Sup
48 h sup
72 h Sup
100
U937
80
% of apoptosis
60
40
20
0
S100A8
Con
Con
S100A8
S100A8
Con
S100A8
Con
Con
24 h Sup
48 h Sup
72 h Sup
100
Jurkat
80
% of apoptosis
60
40
20
0
S100A8
Con
Con
S100A8
S100A8
Con
Con
S100A8
48 h Sup
72 h Sup
Con
24 h Sup
Supplementary Figure 3

## Slide 4
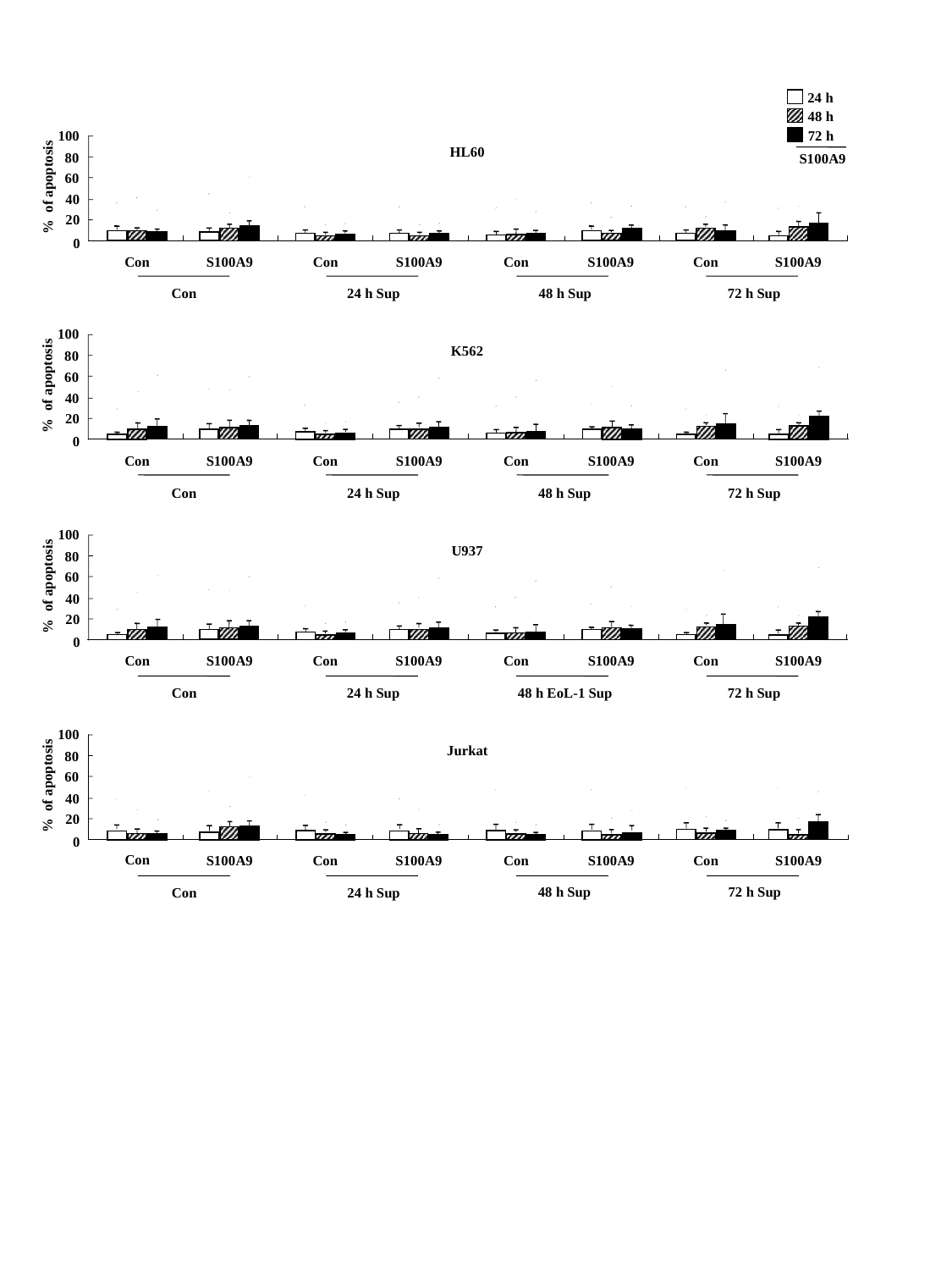

24 h
48 h
72 h
100
HL60
S100A9
80
% of apoptosis
60
40
20
0
S100A9
Con
Con
S100A9
S100A9
Con
S100A9
Con
Con
24 h Sup
48 h Sup
72 h Sup
100
K562
80
% of apoptosis
60
40
20
0
S100A9
Con
Con
S100A9
S100A9
Con
S100A9
Con
Con
24 h Sup
48 h Sup
72 h Sup
100
U937
80
% of apoptosis
60
40
20
0
S100A9
Con
Con
S100A9
S100A9
Con
S100A9
Con
Con
24 h Sup
48 h EoL-1 Sup
72 h Sup
100
Jurkat
80
% of apoptosis
60
40
20
0
Con
S100A9
Con
S100A9
Con
S100A9
S100A9
Con
48 h Sup
72 h Sup
Con
24 h Sup

## Slide 5
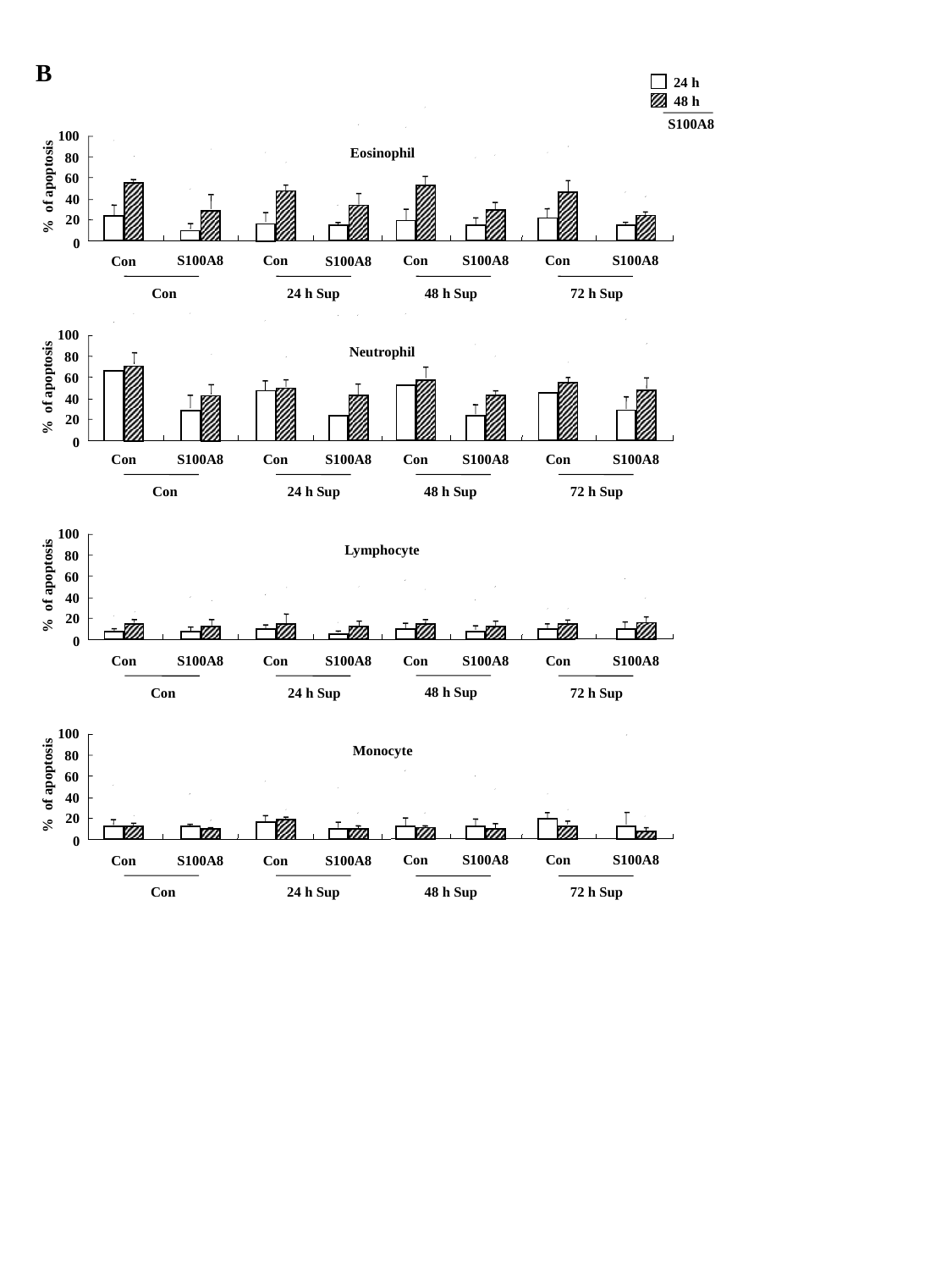

B
24 h
48 h
S100A8
100
Eosinophil
80
% of apoptosis
60
40
20
0
Con
S100A8
S100A8
Con
S100A8
Con
Con
S100A8
Con
24 h Sup
48 h Sup
72 h Sup
100
Neutrophil
80
60
% of apoptosis
40
20
0
S100A8
Con
Con
S100A8
S100A8
Con
S100A8
Con
Con
24 h Sup
48 h Sup
72 h Sup
100
Lymphocyte
80
% of apoptosis
60
40
20
0
S100A8
Con
Con
S100A8
S100A8
Con
S100A8
Con
48 h Sup
Con
24 h Sup
72 h Sup
100
Monocyte
80
% of apoptosis
60
40
20
0
Con
S100A8
S100A8
Con
S100A8
Con
Con
S100A8
Con
24 h Sup
48 h Sup
72 h Sup

## Slide 6
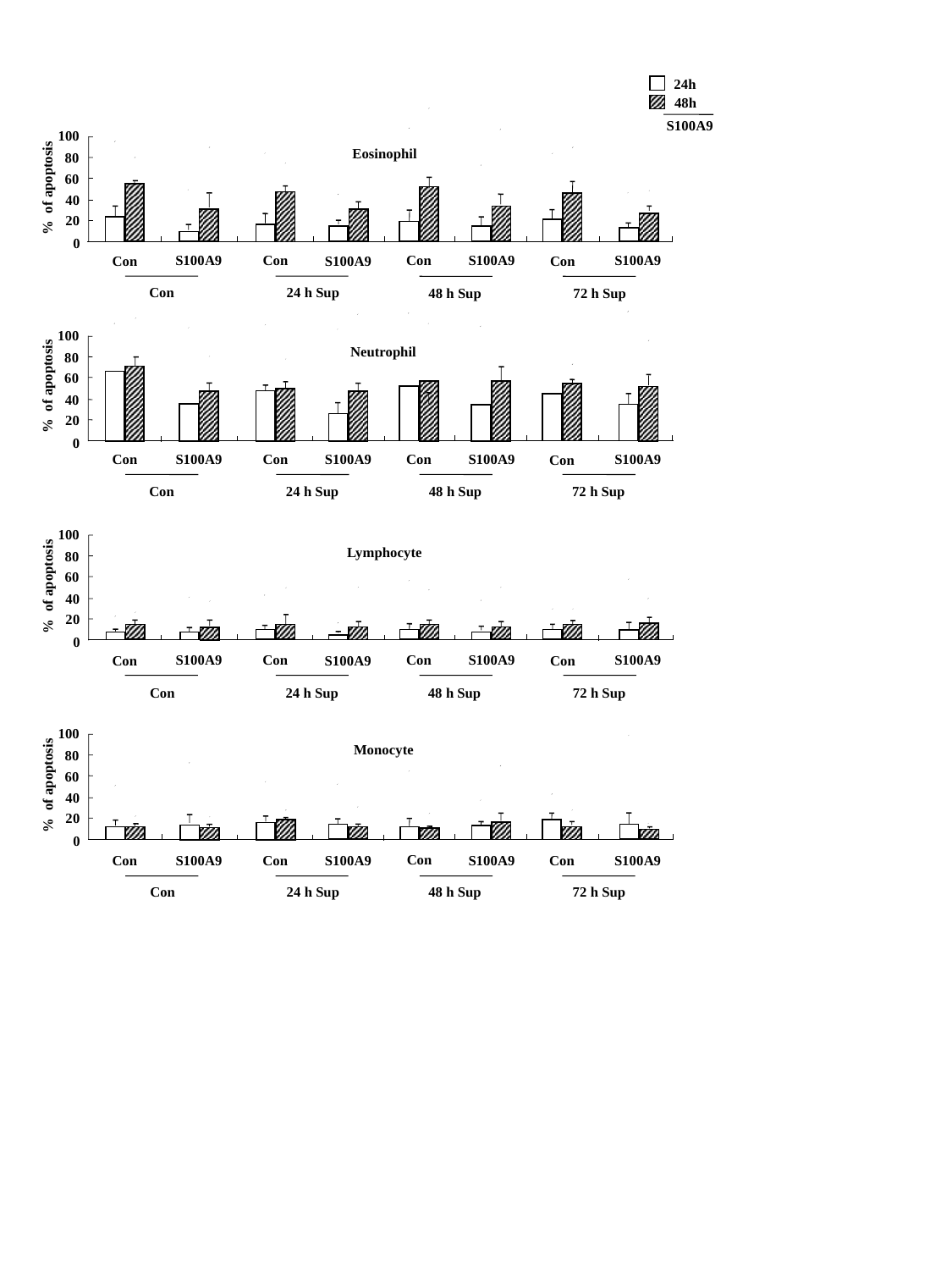

24h
48h
S100A9
100
Eosinophil
80
% of apoptosis
60
40
20
0
S100A9
S100A9
S100A9
Con
Con
Con
S100A9
Con
Con
24 h Sup
48 h Sup
72 h Sup
100
Neutrophil
80
% of apoptosis
60
40
20
0
S100A9
Con
Con
Con
S100A9
S100A9
S100A9
Con
Con
24 h Sup
48 h Sup
72 h Sup
100
Lymphocyte
80
% of apoptosis
60
40
20
0
Con
S100A9
S100A9
Con
S100A9
Con
S100A9
Con
Con
24 h Sup
48 h Sup
72 h Sup
100
Monocyte
80
% of apoptosis
60
40
20
0
Con
Con
S100A9
S100A9
Con
S100A9
S100A9
Con
Con
24 h Sup
48 h Sup
72 h Sup

## Slide 7
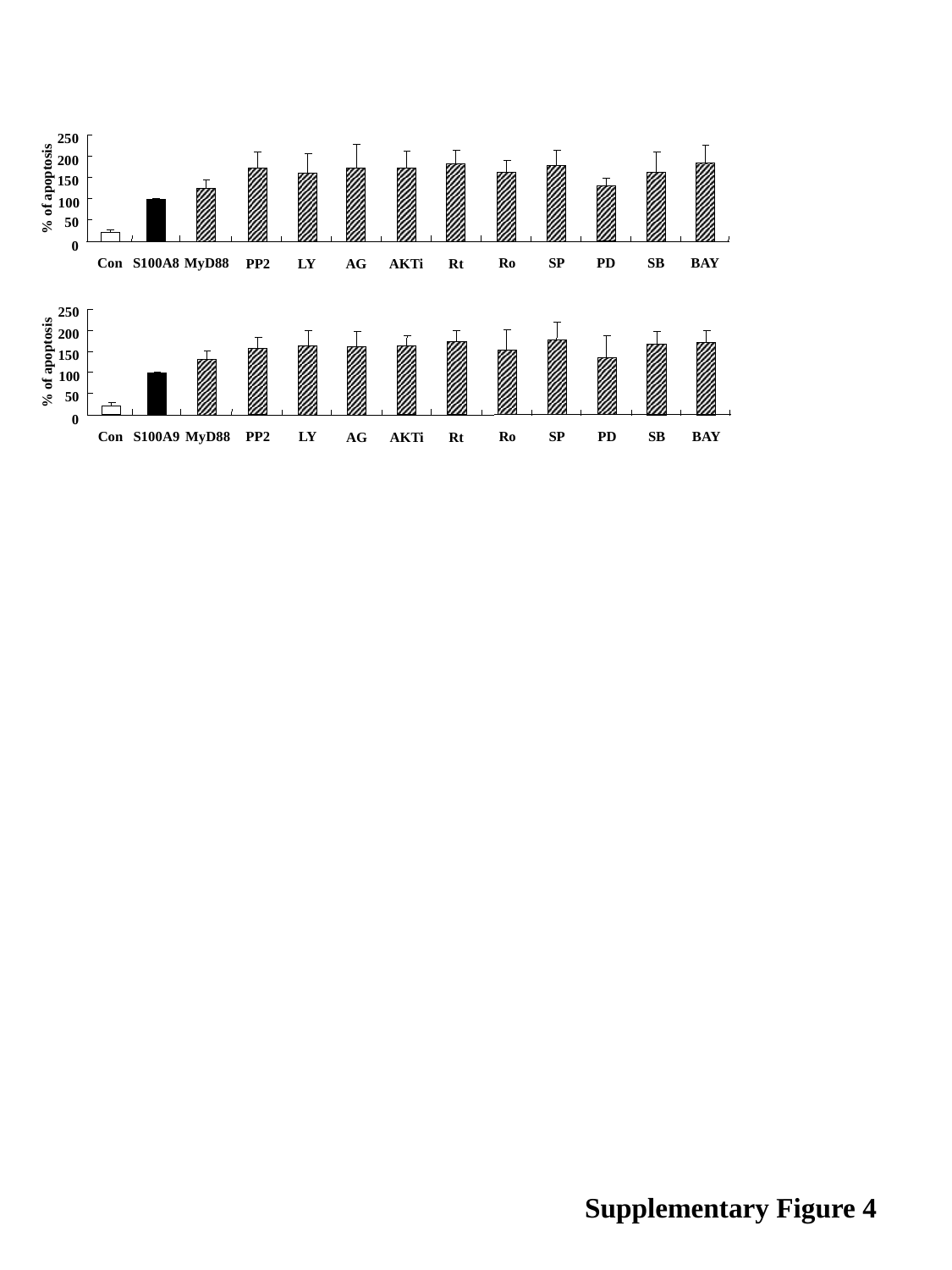

250
200
% of apoptosis
150
100
50
0
MyD88
Con
S100A8
Ro
SP
PD
SB
BAY
PP2
LY
AKTi
Rt
AG
250
200
% of apoptosis
150
100
50
0
S100A9
MyD88
PP2
Ro
SP
PD
SB
BAY
Con
LY
AKTi
Rt
AG
Supplementary Figure 4

## Slide 8
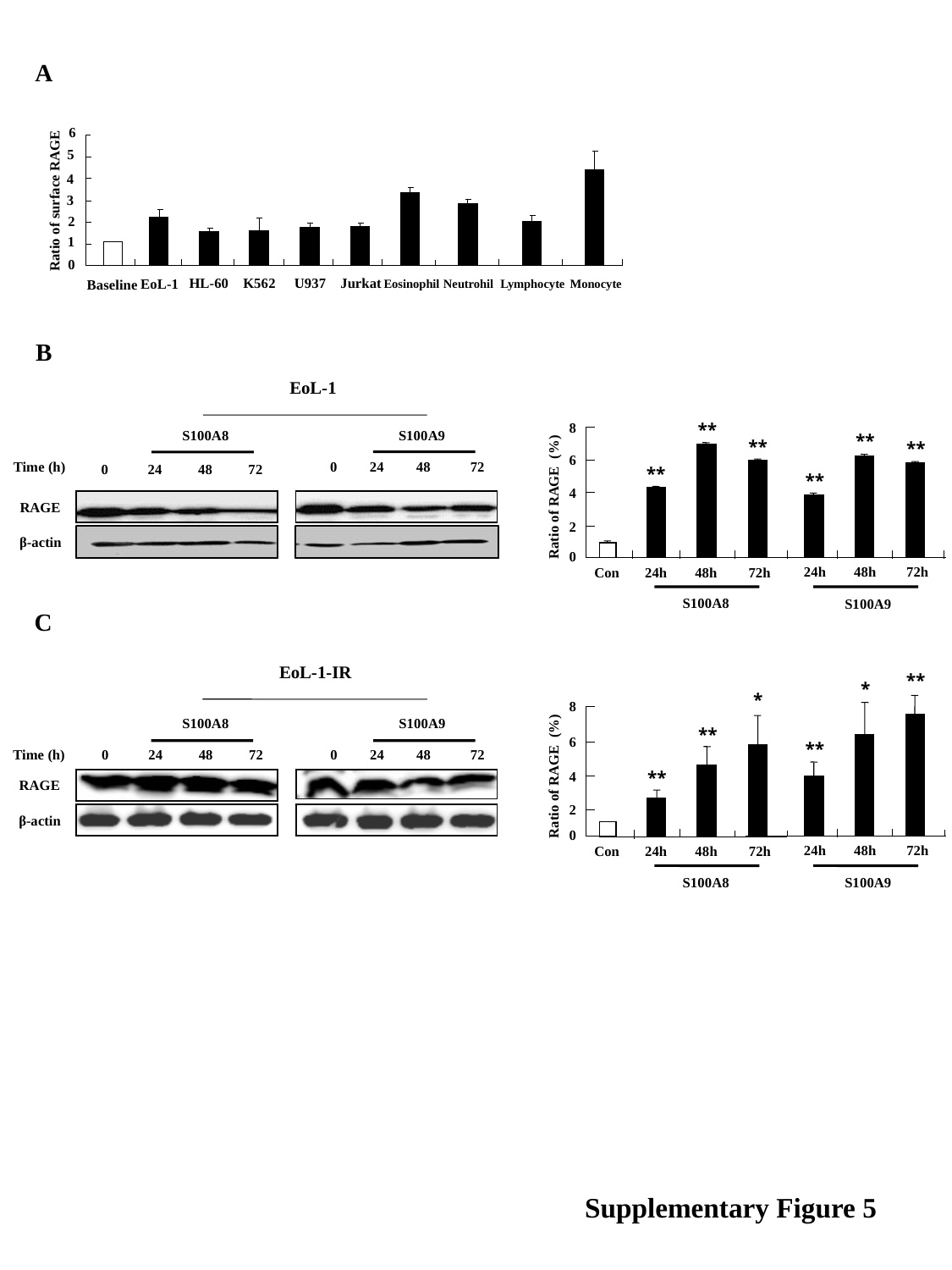

A
6
5
4
Ratio of surface RAGE
3
2
1
0
HL-60
K562
U937
Jurkat
EoL-1
Baseline
Neutrohil
Monocyte
Lymphocyte
Eosinophil
B
EoL-1
**
8
6
Ratio of RAGE (%)
4
2
0
24h
48h
 72h
Con
24h
48h
 72h
S100A8
S100A9
S100A8
S100A9
**
**
**
 0 24 48 72
Time (h)
 0 24 48 72
**
**
RAGE
β-actin
C
EoL-1-IR
**
*
*
8
S100A8
S100A9
**
**
6
Time (h)
 0 24 48 72
 0 24 48 72
Ratio of RAGE (%)
**
4
RAGE
2
β-actin
0
24h
48h
 72h
Con
24h
48h
 72h
S100A8
S100A9
Supplementary Figure 5
